# Inositol-Dependent Alleviation of Lithium-Induced Embryonic Neurotoxicity in Mice Through the GSK3β/β-Catenin/CyclinD1 Pathway

**DOI:** 10.3390/ijms27219785

**Published:** 2026-10-30

**Authors:** Shen Li, Jin Guo, Xiuwei Wang, Zhen Guan, Zhiqiang Zhu, Aiyun Yang, Jianhua Wang

**Affiliations:** Capital Center for Children’s Health, Capital Medical University, Capital Institute of Pediatrics, No. 2 Yabao Road, Chaoyang District, Beijing 100020, China

**Keywords:** inositol, intervention, lithium carbonate, embryonic, neurotoxicity, GSK3β/β-catenin/CyclinD1 pathway

## Abstract

Lithium carbonate (Li_2_CO_3_) is a first-line pharmacotherapy for bipolar disorder (BPD) in female patients of childbearing age, although it is associated with embryonic teratogenicity. Lithium exposure in batteries and pharmaceuticals has been a toxicity factor for women’s reproductive health in energy, environment, and fitness fields. Neural tube defects (NTDs) are the severest reproductive outcome of maternal Li_2_CO_3_ exposure. Currently, there is a lack of effective prevention for Li_2_CO_3_ neurotoxicity. Our previous study revealed that lithium-induced murine NTDs correlated with enhanced proliferation and impaired inositol biosynthesis. In this study, 400 mg/kg inositol was used to treat the lithium-induced murine NTDs after calculating the toxicity using ADMETlab 3.0 and Deep-PK. We found that inositol supplementation ameliorated fetal neural phenotypes, crown-rump length, and body weight and rescued aberrant cell proliferation and dysregulated the GSK3β/β-catenin/CyclinD1 pathway in both animal and cellular models. The affinity of GSK3β-inositol interaction was analyzed and validated using molecular dynamics and surface plasmon resonance. GSK3β-edited cell models further revealed that inositol altered GSK3β post-transcriptional status and phosphorylation, consequently modulating the GSK3β/β-catenin/CyclinD1 signaling cascade. Overall, our study clarified that inositol alleviates lithium-induced NTDs via the GSK3β/β-catenin/CyclinD1 pathway, revealed the direct molecular interaction between inositol and GSK3β, and highlighted the potential of inositol as a therapeutic prevention against lithium-induced embryonic neurotoxicity for childbearing female BPD patients.

## 1. Introduction

Bipolar disorder (BPD) is a general and severe psychosis known worldwide, with an excessive prevalence among women of childbearing age [1]. Lithium carbonate (Li_2_CO_3_) is a first-line pharmacotherapy for the prevention and treatment of BPD [2,3]. Maternal lithium exposure has teratogenic effects on embryonic neurodevelopment in humans and other mammals. The worst outcome of lithium exposure is neural tube defects (NTDs). Balancing BPD treatment and reproductive risk during treatment has become an urgent issue that needs to be addressed. Moreover, the growing environmental concern due to lithium batteries and water pollution from lithium-based pharmaceuticals remains a potential risk [4]. Lithium exposure was reported as a toxicity factor for women’s reproductive health when considering energy, environment, and fitness [5]. The effective prevention of lithium-induced embryonic neurotoxicity has become a key issue.

Researchers and medical professionals are increasingly focusing on the potential of natural compounds to promote health and prevent disease. These compounds are being marketed as nutritional supplements, while others are formulated into various therapeutic drugs [6,7]. The use of natural compounds to reduce the toxic effects of various drugs and environmental pollutants on the human body has also received widespread attention [8]. Inositol, a naturally occurring compound found in several foods, is key to various biological processes. Its derivatives regulate numerous cellular functions, including gene transcription, membrane excitability, vesicular trafficking, calcium signaling, and neuronal development. Inositol deficiency is associated with the prevalence of NTDs [9]. Inositol supplementation might be a viable approach to prevent NTDs. A previous study established a stable lithium-induced murine NTD model and revealed inositol biosynthesis impairment and enhanced cell proliferation in lithium-induced NTDs [10]. To the best of our knowledge, whether inositol could reverse lithium-induced NTDs has not been researched.

In our previous study, analysis of human NTD embryonic brain tissue indicated that inositol depletion might cause upregulation of the PI3K/Akt pathway in the developing neural tube [11]. Glycogen synthase kinase 3β (GSK3β) is located downstream of the PI3K/Akt pathway and is one of the targets of lithium. This was highly expressed in the central nervous system and has been implicated in neural development, neuron polarization, and neurodegenerative diseases [12]. GSK3β-null mice exhibited craniofacial malformation [13]. In vitro research has found that GSK3 was needed for the synthase activity of inositol-3 phosphate and the de novo biosynthesis of inositol. GSK3 inactivation resulted in inositol depletion [14]. Studies have demonstrated that GSK3β phosphorylation modulates cell proliferation when acting as an inhibitor of β-catenin and regulates the occurrence of NTDs [15]. Cell cycle protein D1 (CyclinD1) is a downstream target gene regulated by β-catenin. The GSK3β/β-catenin/CyclinD1 pathway participates in the proliferation of neural progenitor cells, neural stem cells, and oligodendrocytes, thereby affecting neural improvement [16]. To the best of our knowledge, the impact of the GSK3β/β-catenin/CyclinD1 pathway on lithium-induced NTDs has not been previously reported.

In the present study, the aim was to find out whether inositol could alleviate lithium-induced NTDs and assess the impact of inositol on cell proliferation and the GSK3β/β-catenin/CyclinD1 pathway. First, the dynamic expression of GSK3β and its phosphorylation in the lithium-induced NTDs was investigated. Then, the effects of inositol supplementation in lithium-induced neurotoxicity were examined by evaluating the embryonic weight, crown-rump length, and the rate of NTDs. The interaction of inositol and GSK3β was analyzed through molecular simulation and experimental validation. Moreover, the regulation of inositol in cell proliferation and the GSK3β/β-catenin/CyclinD1 pathway were investigated in the murine model and gene-edited cell model. The present study provided novel insight into the nutritional supplementation needs of women of childbearing age with BPD taking Li_2_CO_3_ and laid a foundation for preventing lithium-induced embryonic neurotoxicity.

## 2. Results

### 2.1. The Dynamic Expression of GSK3β and Its Phosphorylation in Lithium-Induced NTDs

**Figure 1:**
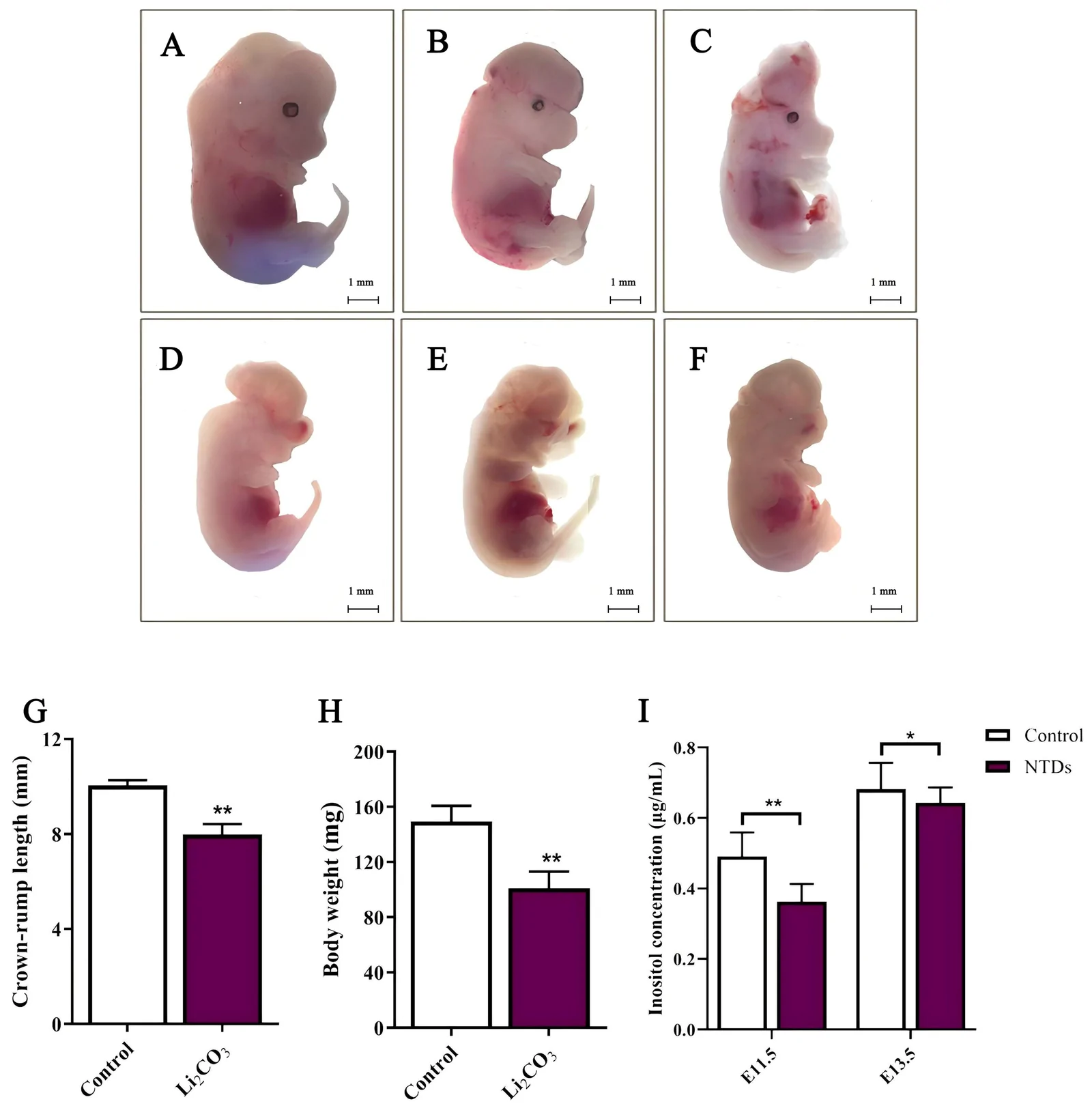
Morphological and developmental changes in normal and abnormal embryos on E13.5 (**A**) The Control embryo under dissecting microscope; (**B**–**F**) NTD embryos post Li_2_CO_3_ treatment under dissecting microscope: (**B**–**D**) encephalocele; (**E**,**F**) craniofacial malformation; (**G**) the crown-rump length of Control and NTD group; (**H**) The body weight of Control and NTD group; (**I**) The inositol levels of embryonic neural tissues in Control and NTD groups on E11.5 and E13.5. * *p* < 0.05 vs. Control group. ** *p* < 0.01 vs. Control group. Data are shown as mean ± SD (*n* = 12). Scale bar = 1 mm.

In our previous study, a stable lithium-induced NTD murine model was established [10]. The development of the neural tube was observed at E13.5 under stereoscopy. The neural tube was smooth and flat, with complete closure in the Control embryos. By contrast, an unclosed neural tube and NTD phenotypes, such as encephalocele, were observed in the Li_2_CO_3_ group (Figure 1A–F). The average occurrence rate of NTDs was 33.3% in the Li_2_CO_3_ group (Table 1). The average crown-rump length (CRL) of the Control embryos was 10.1 mm, whereas that of the Li_2_CO_3_-treated embryos was 7.9 mm (Figure 1G). The average body weight in the Li_2_CO_3_ group was 101 mg, which was considerably lower than the average of 149.3 mg in the Control group (Figure 1H, *p* < 0.01). Maternal Li_2_CO_3_ exposure affected embryonic growth and development, especially embryonic neurodevelopment. Inositol levels were examined in the neural tissues of Control and NTD (lithium-induced NTDs) embryos on E11.5 and E13.5. Inositol levels were notably decreased within the NTDs when compared with those in the Control (Figure 1I).

**Figure 2:**
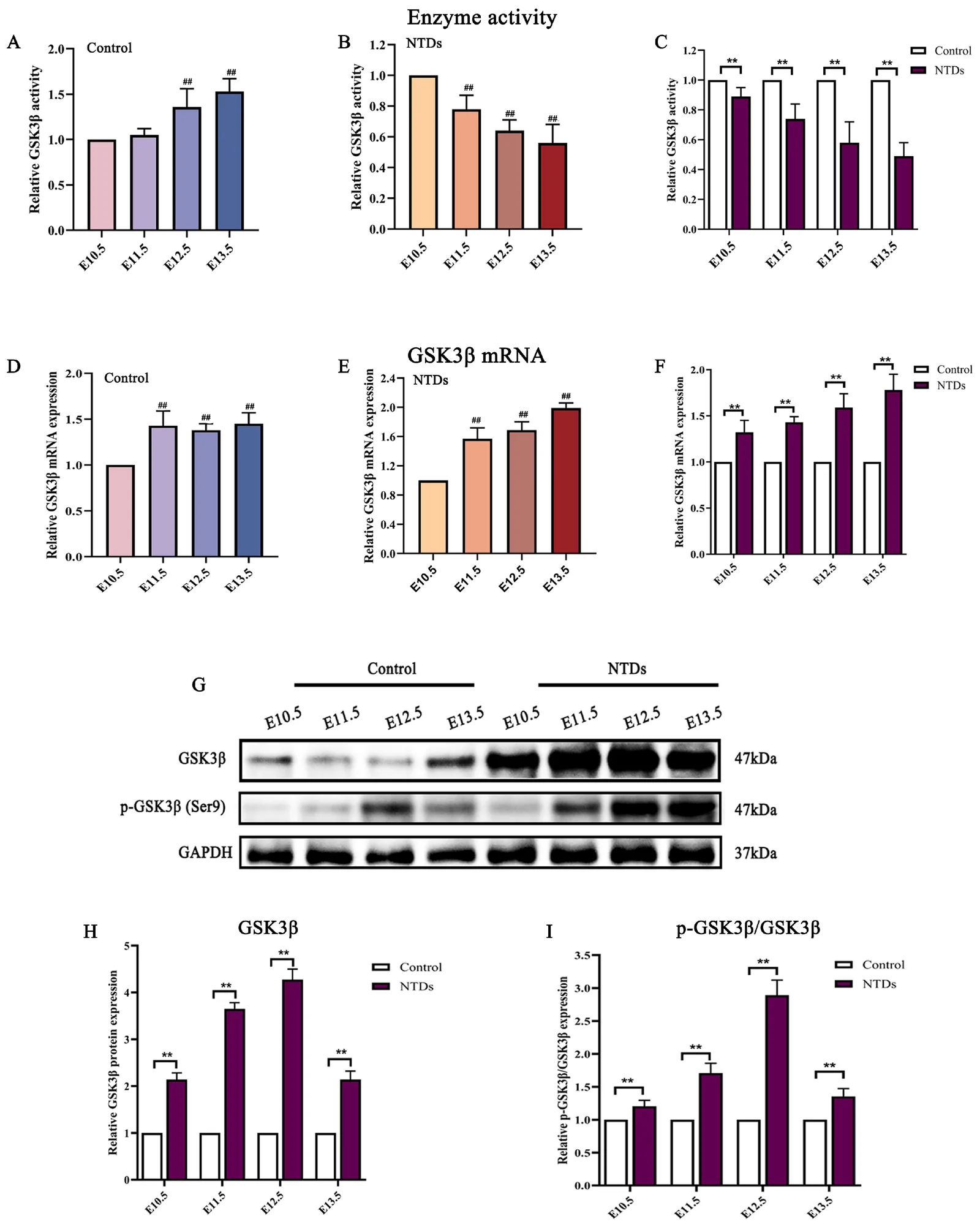
The dynamic expression of GSK3β and its phosphorylation in lithium-induced NTDs during E10.5–E13.5. (**A**,**B**) The enzyme activity of GSK3β in embryonic neural tissues of Control and NTD group; (**C**) the comparison of GSK3β enzyme activity between Control group and NTD group at E10.5–E13.5; (**D**,**E**) the mRNA expression of GSK3β in embryonic neural tissues of Control and NTD group; (**F**) the comparison of GSK3β mRNA expression between Control group and NTD group at E10.5–E13.5; (**G**) Western blot of the protein levels of GSK3β and its phosphorylation in embryonic neural tissues of Control and NTD group at E10.5–E13.5. GAPDH was used as a loading Control. (**H**,**I**) Quantification results of GSK3β and its phosphorylation in (**G**). ^##^ *p* < 0.01 vs. E10.5, ** *p* < 0.01 vs. Control group. Data are shown as mean ± SD (*n* = 3).

**Figure 3:**
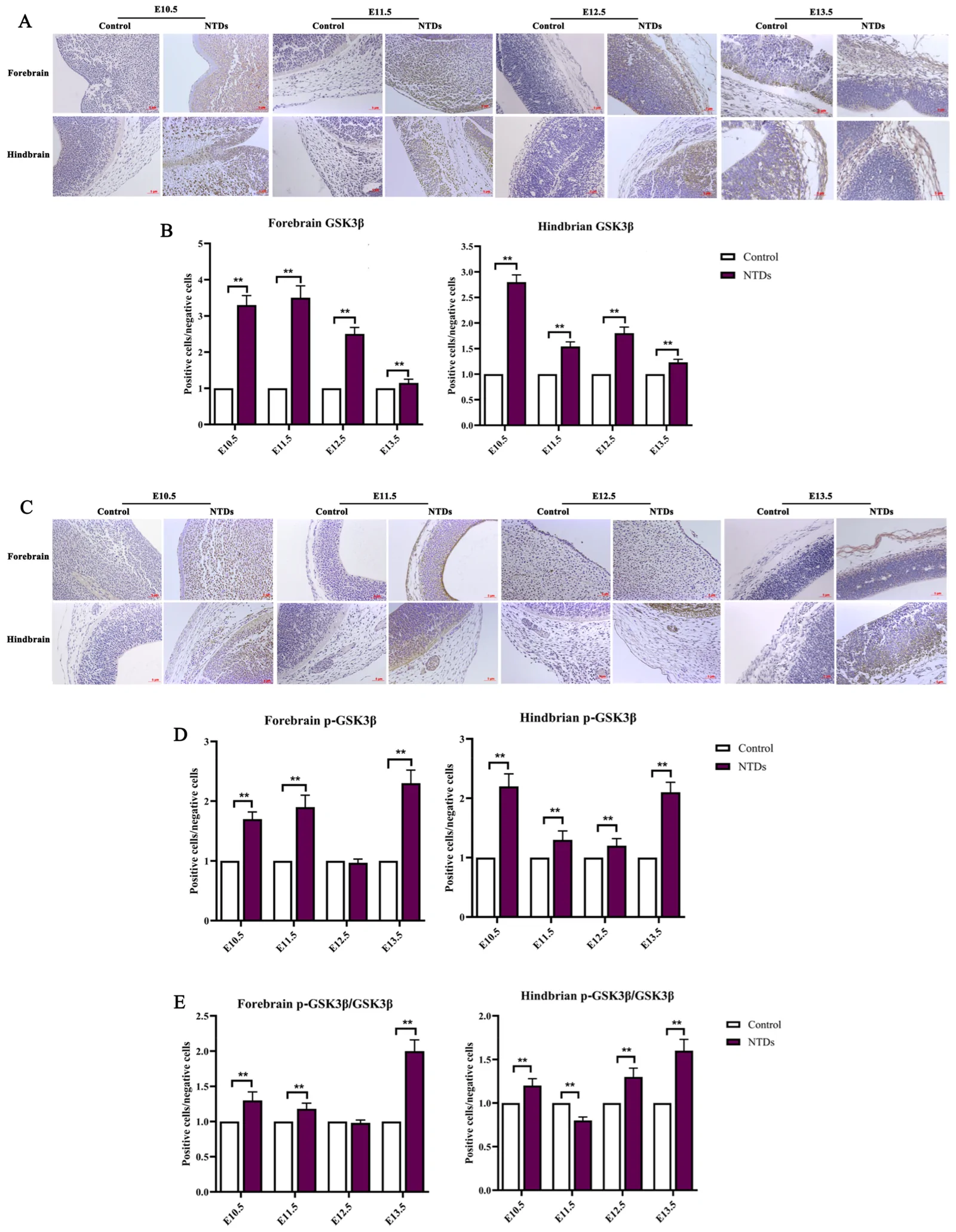
Immunohistochemistry of the dynamic protein expression of GSK3β and its phosphorylation in the embryonic forebrain and hindbrain of lithium-induced NTDs during E10.5–E13.5. (**A**) The comparison of GSK3β expression between Control group and NTD group at E10.5–E13.5; (**B**) quantification result of GSK3β in forebrain and hindbrain; (**C**) the comparison of phospho-GSK3β expression between Control group and NTD group at E10.5–E13.5; (**D**) quantification result of phospho-GSK3β in forebrain and hindbrain; (**E**) quantification result of GSK3β phosphorylation in forebrain and hindbrain between Control group and NTD group at E10.5–E13.5. ** *p* < 0.01 vs. Control group. Data are shown as mean ± SD (*n* = 3).

The dynamic expression and enzyme activity level of GSK3β in embryonic neural tissues were evaluated from E10.5 to E13.5. The enzyme activity of GSK3β in the Control group stabilized at E10.5–E11.5 and accelerated at E12.5–E13.5. Within the NTD group, GSK3β activity constantly decreased between E10.5 and E13.5 (Figure 2A–C). The qRT-PCR effects prolonged GSK3β mRNA expression between E10.5 and E11.5 and stabilized it between E12.5 and E13.5 within the Control group. A continued increase in GSK3β mRNA expression in the NTD group was observed, which was greater than that in the Control group (Figure 2D–F). The phosphorylation of GSK3β was significantly higher in the NTD group when compared with that in the Control group (Figure 2G–I). Immunohistochemical analysis showed increased GSK3β expression in the forebrain and hindbrain of the NTD group during the E10.5–E13.5 period, in contrast to the Control group (Figure 3A,B). At E10.5, E11.5, and E13.5, phospho-GSK3β expression was increased in the forebrain and hindbrain of the NTDs compared with those of the Control (Figure 3C,D). The phosphorylation of GSK3β within the forebrain and hindbrain was substantially greater in the NTDs compared with the Control (Figure 3E). Maternal Li_2_CO_3_ exposure induced inositol depletion in embryonic neural tissue, resulting in decreased enzyme activity due to increased expression of GSK3β and its inhibitory phosphorylation.

### 2.2. Role of Inositol on Lithium-Induced NTDs and GSK3β/β-Catenin/CyclinD1 Pathway at E13.5

Inositol deficiency caused by maternal Li_2_CO_3_ exposure led to NTDs. Pregnant mice exposed to Li_2_CO_3_ were treated with inositol to clarify whether inositol supplementation could rescue lithium-induced NTDs. Prediction of toxicity was needed prior to inositol intervention to prevent cumulative teratogenic effects of Li_2_CO_3_. The ideal drug should exhibit no toxicity and be eliminated without affecting its biological activity. Consequently, the focus was on the toxicity of Li_2_CO_3_ and inositol through calculations using ADMETlab 3.0 and Deep-PK. The results are presented in Table 2. Li_2_CO_3_ was calculated to potentially be toxic in the regions of drug-induced liver injury, skin sensitization, carcinogenicity, eye corrosion, eye irritation, human hepatotoxicity, hematotoxicity, genotoxicity, drug-induced neurotoxicity, micronuclei, and estrogen receptors, whereas inositol showed no toxicity in these metrics. These findings suggested that treatment with inositol may mitigate lithium-induced toxicity. These consequences indicated low toxicity levels, positioning inositol as a promising candidate for the treatment of lithium-precipitated NTDs.

**Figure 4:**
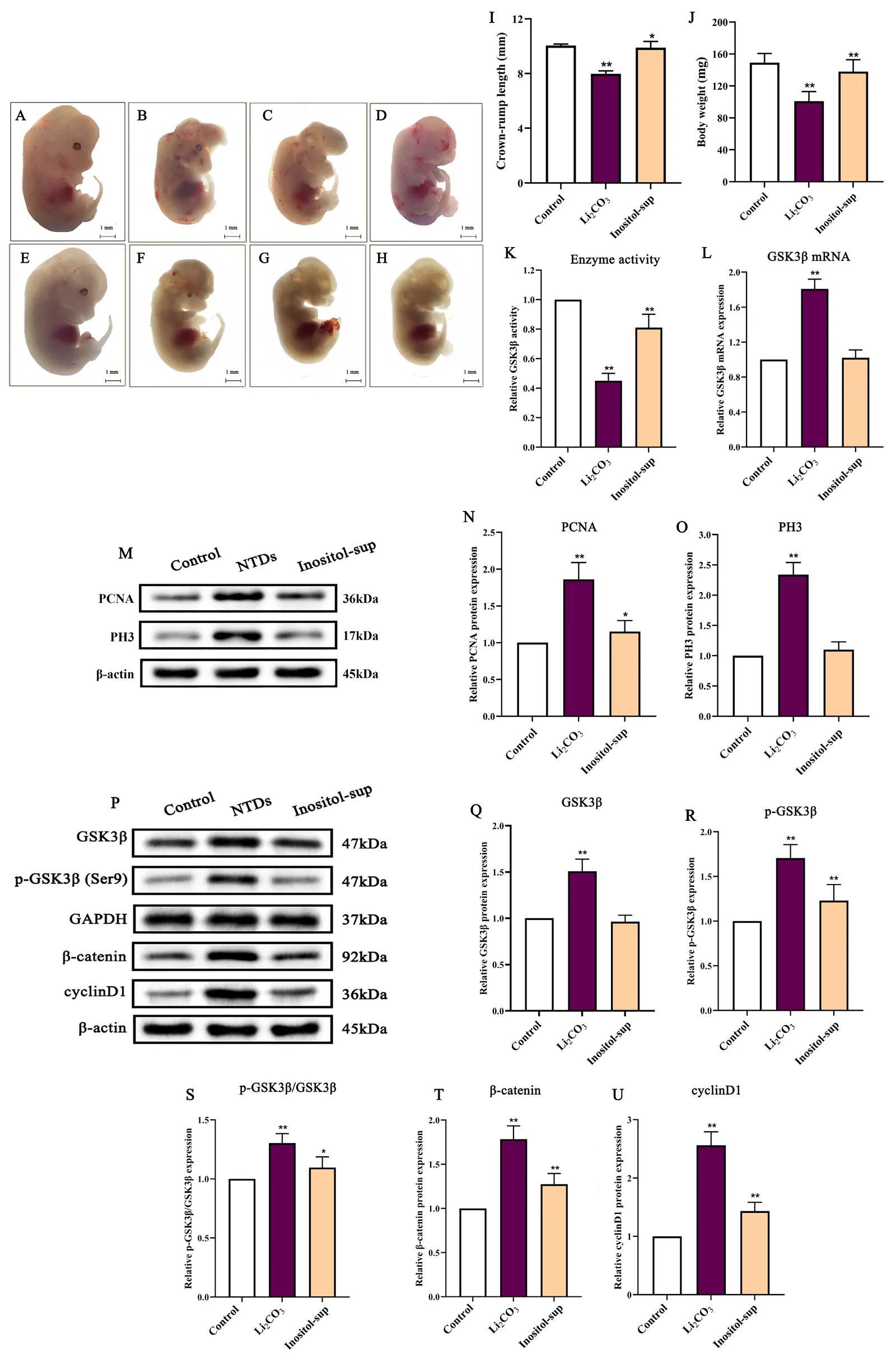
The developmental changes and GSK3β/β-catenin/CyclinD1 pathway in lithium-treated embryos with inositol supplementation at E13.5. (**A**) The Control embryo under dissecting microscope; (**B**–**D**) NTD embryos post Li_2_CO_3_ treatment under dissecting microscope: (**B**) encephalocele; (**C**,**D**) craniofacial malformation; (**E**–**H**) the normal and abnormal embryos after inositol supplementation under dissecting microscope: (**E**) the normal embryos after inositol supplementation; (**F**–**H**) the abnormal embryos after inositol supplementation: (**F**) encephalocele; (**G**,**H**) craniofacial malformation; (**I**,**J**) the crown-rump length and body weight of Control, NTD and Inositol-sup group (*n* = 12); (**K**,**L**): the enzyme activity and mRNA expression of GSK3β in embryonic neural tissues of Control, NTD and Inositol-sup group; (**M**) Western blot of PCNA and PH3 expression in embryonic neural tissues of Control, NTD and Inositol-sup group; (**N**,**O**) quantification result of PCNA and PH3 in (**M**); (**P**) Western blot of GSK3β/β-catenin/CyclinD1 pathway expression in embryonic neural tissues of Control, NTD and Inositol-sup group. β-actin was used as loading Control; (**Q**–**U**) Quantification result of GSK3β, phospho-GSK3β, β-catenin and CyclinD1 in (**P**). * *p* < 0.05 vs. Control group. ** *p* < 0.01 vs. Control group. Data are shown as mean ± SD (*n* = 3).

In a previous study, Li_2_CO_3_-treated pregnant mice were supplemented with 400 mg/kg inositol on E7.5 and dissected on E13.5 to observe the embryonic development of the mice after inositol supplementation. The proportion of phenotypically normal embryos in these mice increased substantially (from 20.6 to 60.8%). The incidence of resorbed embryos and NTDs substantially decreased (*p* < 0.01), with the incidence of NTDs decreasing from 33.3 to 14.9% and the percentage of resorbed embryos decreasing from 7.9 to 1.4%. The incidence of ocular anomalies markedly decreased (Table 3). Notably, there was no phenotype of growth retardation in the embryos after inositol supplementation. The severity of the embryonic malformations was mitigated after inositol supplementation, and a comparison of embryo phenotypes before and after inositol supplementation is shown in Figure 4A–H. As shown in Figure 4I,J, inositol supplementation improved the CRL and body weight of Li_2_CO_3_-treated embryos to some extent.

The cell proliferation markers (PH3 and PCNA) were detected in embryonic neural tissues from the Control, NTD, and Inositol-sup (normal embryos after inositol supplementation) groups. Compared with the NTDs, inositol supplementation reduced the expression levels of PH3 and PCNA, which indicated that abnormal cell proliferation was alleviated (Figure 4M–O). GSK3β enzyme activity and expression were further examined in embryonic neural tissues, which revealed that inositol supplementation restored GSK3β enzyme activity to a certain extent (Figure 4K) and rescued the lithium-induced expression of GSK3β and its high level of inhibitory phosphorylation (Figure 4L,P–S). Expression of β-catenin and CyclinD1 was further tested, which are regulated by GSK3β phosphorylation, suggesting that inositol supplementation would attenuate the activated GSK3β/β-catenin/CyclinD1 signaling pathway due to maternal Li_2_CO_3_ exposure (Figure 4P–U).

### 2.3. Molecular Simulation and Validation of the Inositol and GSK3β Interaction

**Figure 5:**
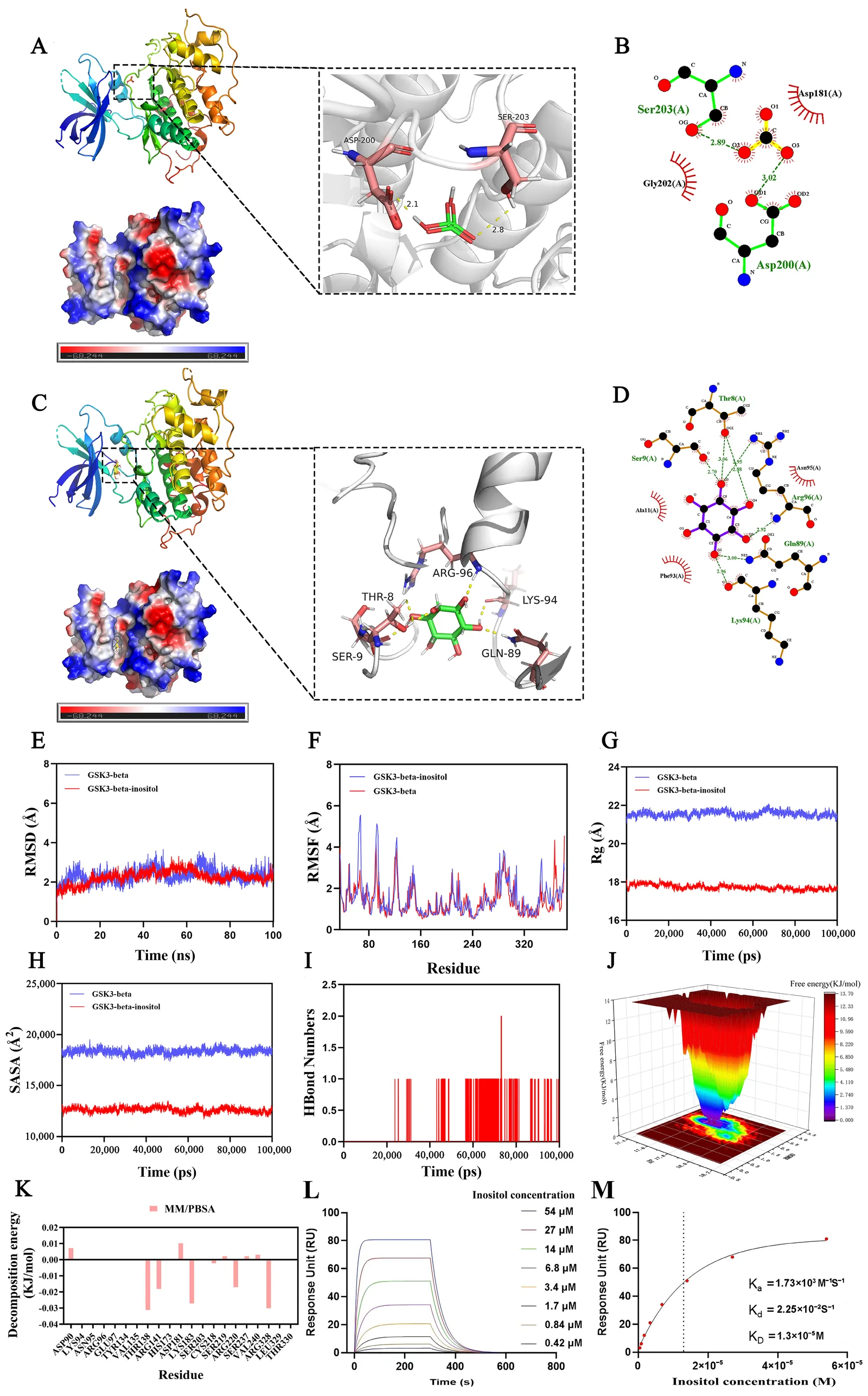
Molecular simulation and validation of the inositol and GSK3β interaction. (**A**,**B**) Molecular docking analysis of GSK3β and Li_2_CO_3_; (**C**,**D**) molecular docking analysis of GSK3β and inositol; (**A**,**C**): rainbow cartoon: blue (N-terminus) to red (C-terminus); yellow sticks = ligand. Electrostatic surface: red = negative potential, blue = positive potential, yellow sticks = ligand. (**B**,**D**): Yellow: carbon atoms of the ligand; Green: carbon atoms of protein residues; Red: oxygen atoms; Blue: nitrogen atoms. Green dashed lines: hydrogen bonds (distances in Å). Red arcs with spokes: hydrophobic contacts. (**E**) the root mean square deviation (RMSD) of GSK3β and GSK3β-inositol complex; (**F**) the root mean square function (RMSF) of GSK3β and GSK3β-inositol complex; (**G**) the radius of gyration (Rg) of GSK3β and GSK3β-inositol complex; (**H**) the solvent-accessible surface area (SASA) of GSK3β and GSK3β-inositol complex; (**I**) the hydrogen bond analysis of GSK3β-inositol complex; (**J**) the free energy landscape of GSK3β-inositol complex; (**K**) the binding amino acids analysis of GSK3β-inositol complex. (**L**,**M**) The binding and dissociation curves between GSK3β protein and inositol detected by SPR.

To investigate whether GSK3β potentially interacts with Li_2_CO_3_ and inositol, molecular docking and molecular dynamics were used to analyze the strength, site, and stability of binding. Generally, binding energies <−5 kcal/mol are considered to indicate precise binding between a ligand and a receptor. The binding energies of GSK3β with Li_2_CO_3_ and inositol were −3 and −7 kcal/mol, respectively. The molecular docking consequences revealed that GSK3β had more binding affinity for inositol than for Li_2_CO_3_. This may be due to the strong intermolecular hydrogen bonding interactions between inositol and the residues threonine (THR)-8, serine (SER)-9, glutamine (GLN)-89, lysine (LYS)-94, and arginine (ARG)-96 in the GSK3β protein, at the same time as hydrophobic interactions made through the residues alanine (ALA)-1, phenylalanine (PHE)-93, and asparagine (ASN)-95 (Figure 5C,D). The hydrogen bonding interactions between Li_2_CO_3_ and SER-203 and aspartic acid (ASP)-200 residues and the hydrophobic interactions with the ASP-181 and glycine (GLY)-202 residues within the GSK3β protein are shown in Figure 5A,B.

To further explore the binding balance, MD simulations were carried out on the GSK3β protein and the GSK3β-inositol complex. The model was constructed using the Gromacs 2022 software application, a powerful tool for MD simulations. It was then subjected to a 100 ns equilibration simulation in an aqueous environment to ensure stability and proper system dynamics under the specified conditions. The basis mean RMSD values determined that the GSK3β protein and the GSK3β-inositol complex machine reached equilibrium at ~20 ns and maintained generally low fluctuation tiers, indicating strong binding between GSK3β and inositol over time (Figure 5E). The results implied that RMSF values of the GSK3β-inositol complex system were low (commonly >4 Å), which suggested that they were less flexible and more stable (Figure 5F). The radius of gyration (Rg) and solvent-accessible surface area (SASA) of the GSK3β protein and the GSK3β-inositol complex system fluctuated stably, indicating that the complex of inositol with the GSK3β protein no longer went through adequately sized contraction and enlargement within the path of MD simulation (Figure 5G,H). Compared with those of the GSK3β protein, the Rg and SASA values of the GSK3β-inositol complex system were lower, which suggested a strong binding affinity between inositol and the GSK3β protein. The consequences from the hydrogen bond analysis revealed that the majority of the hydrogen bonds between GSK3β and inositol were 2 during the MD simulation (Figure 5I). These findings indicated that inositol should stably bind to the GSK3β protein and that hydrogen bonds were indispensable to the binding conformation. The MM/PBSA approach was used to calculate the binding free energies of GSK3β and inositol. The binding free energy of the GSK3β-inositol complex system was −18.647 kJ/mol. Negative values indicate molecular binding affinity, and lower values indicate stronger binding. The GSK3β-inositol complex system had high affinity. The distribution of free energy, as derived from the RMSD and Rg calculations, is shown in Figure 5J. Further calculations were performed to analyze the amino acids that contribute to inositol binding in the complex system. The results revealed that THR-138, ARG-328, LYS-183, ARG-220, and ARG-138 had high contribution values in the complex system (Figure 5K). These amino acids may perform important roles in the catalytic process. Based on the findings from molecular docking and molecular dynamics, GSK3β appeared to exhibit a strong binding affinity for inositol. Consequently, molecular interactions between them were validated using SPR analysis. The SPR results indicated that different concentrations of inositol produced distinct binding signals with GSK3β. The response signal saturated and stabilized at ~80 Ru with increasing inositol concentration. (Figure 5L). As shown in Figure 5M, the association and dissociation rates (Ka and Kd) were determined to be 1.73 × 10^3^ M^−1^s^−1^ and 2.25 × 10^−2^ s^−1^, respectively. The calculated affinity (KD) between the GSK-3β protein and inositol was 1.30 × 10^−5^ (M).

### 2.4. Role of Inositol on Lithium-Treated Cell Proliferation and GSK3β/β-Catenin/CyclinD1 Pathway in mEFCs and mNSCs

**Figure 6:**
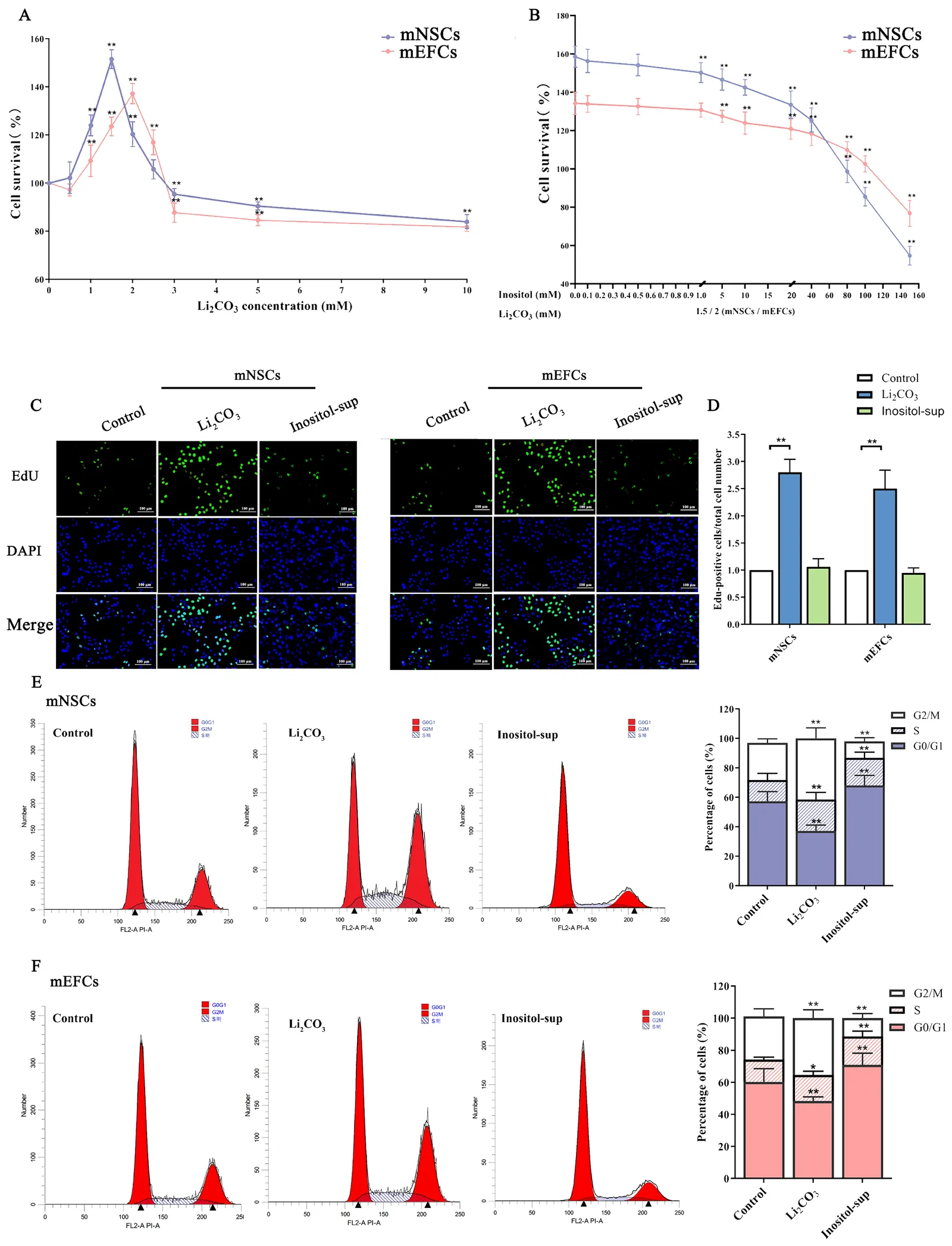
The role of inositol on cell proliferation and cell cycle in lithium-treated cells. (**A**) Cell survival rate of mNSCs and mEFCs treated with different doses of Li_2_CO_3_ for 24 h; (**B**) cell survival rate of lithium-treated mNSCs and mEFCs with different doses of inositol treatment for 24 h; (**C**,**D**) cell proliferation analysis of Control, Li_2_CO_3_, and Inositol-sup group in mNSCs and mEFCs. The scale bar is 100 μm; (**E**,**F**) cell cycle analysis of Control, Li_2_CO_3_, and Inositol-sup groups in mNSCs and mEFCs. * *p* < 0.05, ** *p* < 0.01 vs. Control group. Data are shown as mean ± SD (*n* = 3).

To investigate the effect of Li_2_CO_3_ on cell survival, Li_2_CO_3_ was selected at 0, 0.5, 1, 1.5, 2, 2.5, 3, 5, and 10 mM to treat mouse neural stem cells (mNSCs; NE4Cs cell line) and mouse embryonic fibroblast cells (mEFCs; NIH3T3 cell line), respectively. The MTT assay results after 24 h of Li_2_CO_3_ treatment are shown in Figure 6A. Low concentrations of Li_2_CO_3_ promoted cell survival (*p* < 0.01), whereas high concentrations inhibited survival (*p* < 0.01). Overall, Li_2_CO_3_ had a stronger pro-survival effect on mNSCs when compared with mEFCs. The concentration that produced the highest viability in each cell type after Li_2_CO_3_ treatment was selected to treat the two cell types (1.5 mM for mNSCs and 2 mM for mEFCs) for the selection of inositol supplementation concentration, cell proliferation, and cell cycle detection. Different concentrations of inositol were used to supplement Li_2_CO_3_-treated mNSCs and mEFCs, respectively. As shown in Figure 6B, 80 and 100 mM were selected as the appropriate concentrations of inositol supplementation for mNSCs and mEFCs, respectively. The Control was treated with the same volume of PBS. The EdU results showed increased levels of EdU-positive cells in the Li_2_CO_3_-treated group compared with the Control (*p* < 0.01), specifically inside the mNSCs (Figure 6C,D). Cell cycle detection using flow cytometry revealed a significant reduction in G1-phase cells and an increase in S- and G2/M-phase cells after Li_2_CO_3_ treatment (*p* < 0.01; Figure 6E,F). Low-concentration Li_2_CO_3_ treatment increased cell survival, reduced the number of G1-phase cells, and promoted cell proliferation, which was consistent with the findings of lithium-induced abnormal proliferation in previous animal experiments (10). The pro-proliferative effect of Li_2_CO_3_ on mNSCs was more pronounced when compared with that on mEFCs. The abnormal cell proliferation induced by Li_2_CO_3_ was alleviated with inositol supplementation.

**Figure 7:**
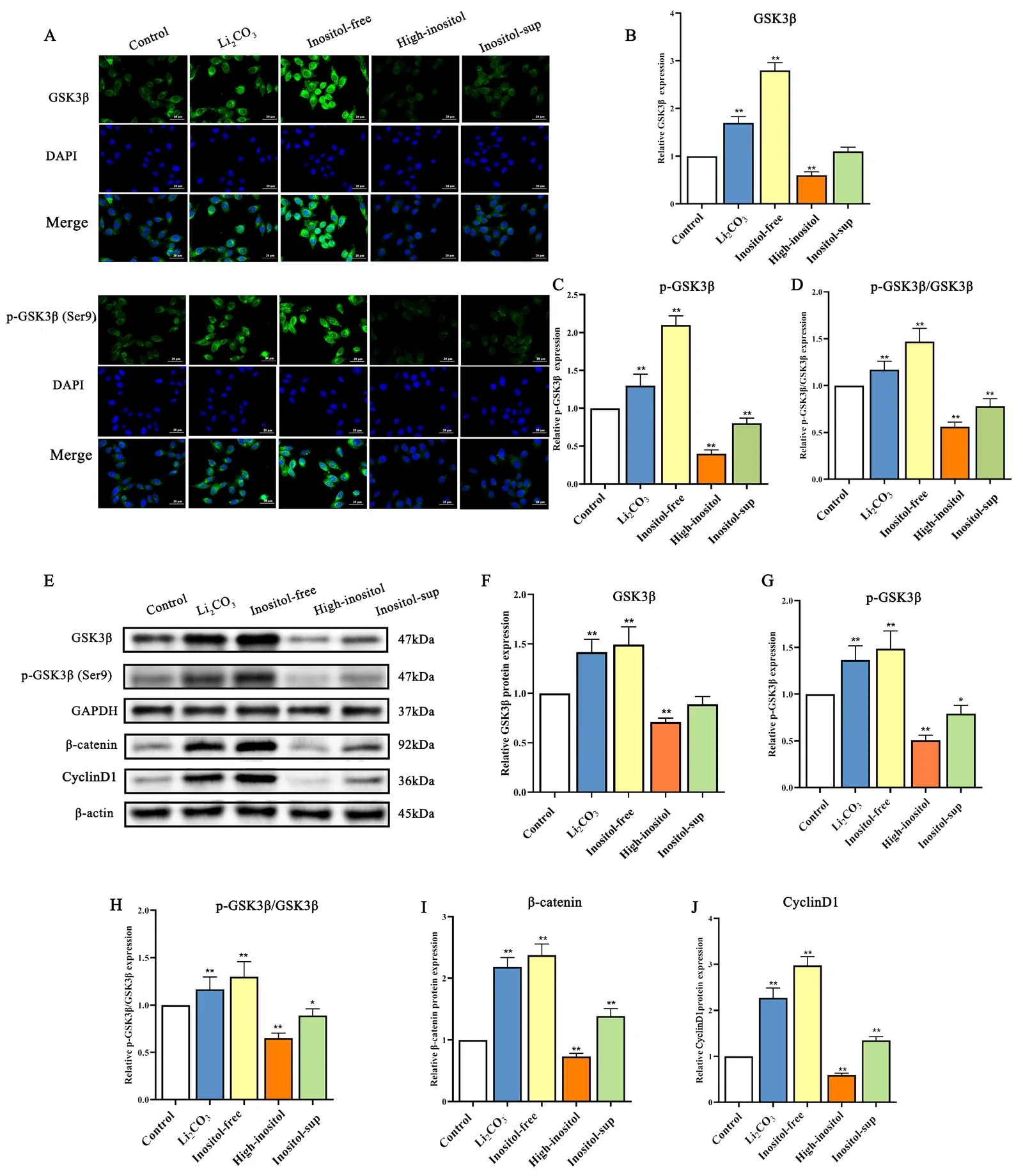
The role of inositol on GSK3β/β-catenin/CyclinD1 pathway in lithium-treated cells. (**A**–**D**) Immunofluorescence assay of the protein levels of GSK3β and its phosphorylation in the Control (PBS), Li_2_CO_3_ (1.5 mM Li_2_CO_3_), Inositol-free (0 mM inositol), High-inositol (80 mM inositol), and Inositol-sup (1.5 mM Li_2_CO_3_ + 80 mM inositol) groups in mNSCs. Confocal images of mNSCs labeled with GSK3β or phospho-GSK3β antibody (green) and cell nucleus (DAPI, blue). The scale bar is 20 μm; (**E**) Western blot of GSK3β/β-catenin/CyclinD1 pathway expression in the Control, Li_2_CO_3_, Inositol-free, High-inositol, and Inositol-sup groups in mNSCs. β-actin was used as loading Control; (**F**–**J**) quantification result of GSK3β, phospho-GSK3β, β-catenin and CyclinD1 in (**E**). * *p* < 0.05, ** *p* < 0.01 vs. Control group. Data are shown as mean ± SD (*n* = 3).

The mNSCs were treated with various concentrations of inositol to measure the levels of GSK3β and its phosphorylation, which were divided into five groups: Control (PBS), Li_2_CO_3_ (1.5 mM Li_2_CO_3_), Inositol-free (0 mM inositol), High-inositol (80 mM inositol), and Inositol-sup (1.5 mM Li_2_CO_3_ + 80 mM inositol) groups. Western blotting and immunofluorescence results revealed that lower inositol levels led to increased GSK3β inhibitory phosphorylation, whereas higher levels hindered this process (Figure 7A–H). Inositol supplementation reversed the activated GSK3β/β-catenin/CyclinD1 pathway (Figure 7E–J).

### 2.5. Role of Inositol on the Regulation of GSK3β/β-Catenin/CyclinD1 Pathway in mNSCs

**Figure 8:**
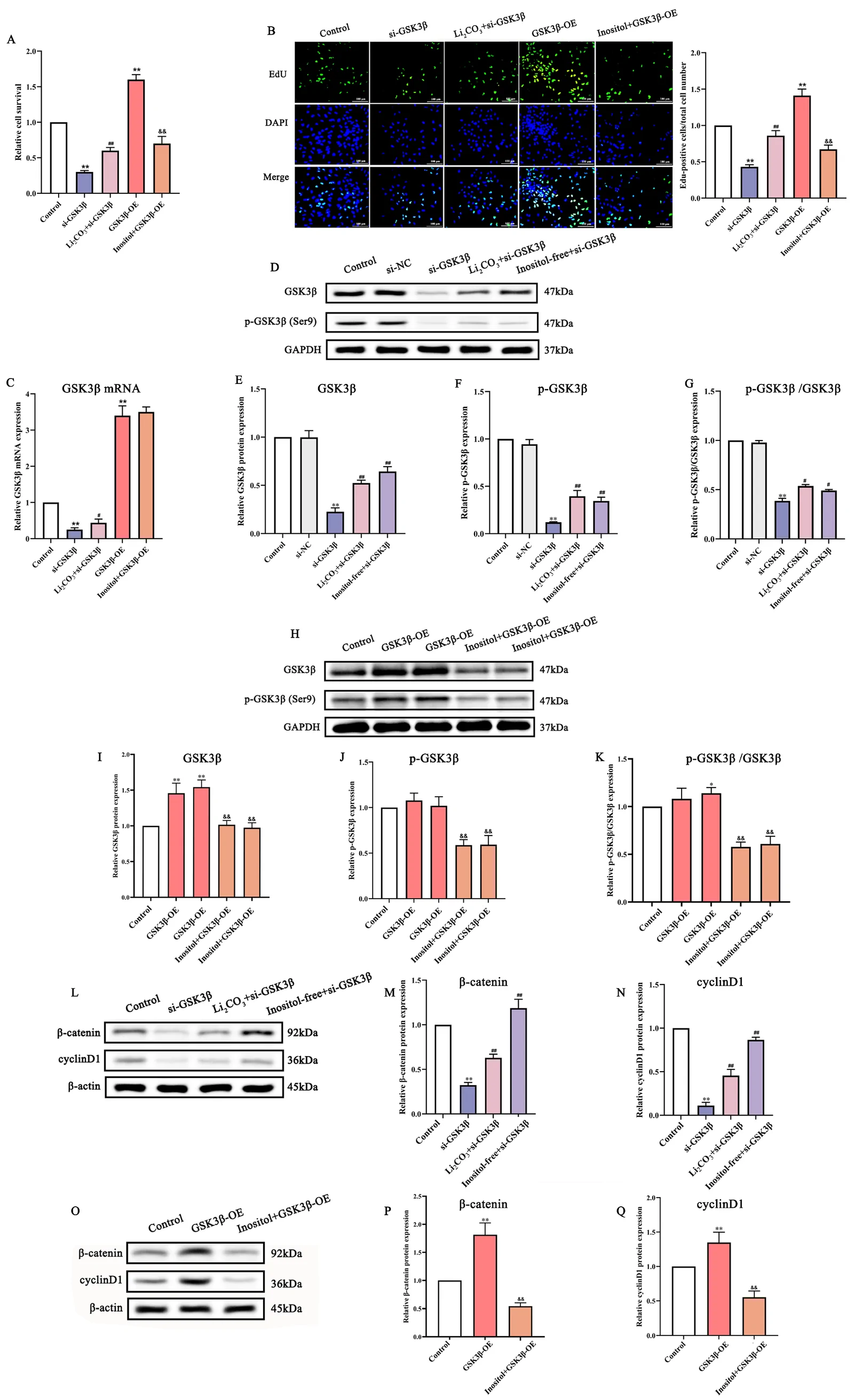
The role of inositol in the regulation of GSK3β/β-catenin/CyclinD1 pathway in *GSK3β*-edited cells. (**A**) Cell survival rate of Control, si-GSK3β, Li_2_CO_3_ + si-GSK3β, GSK3β-OE and Inoistol + GSK3β-OE group; (**B**) cell proliferation analysis of Control, si-GSK3β, Li_2_CO_3_ + si-GSK3β, GSK3β-OE and Inoistol + GSK3β-OE group. The scale bar is 100 μm; (**C**) the mRNA expression of GSK3β of the above five groups; (**D**) Western blot of the protein levels of GSK3β, p-GSK3β of Control, si-NC, si-GSK3β, Li_2_CO_3_ + si-GSK3β and Inositol-free + si-GSK3β group; (**E**–**G**) quantification result of GSK3β and its phosphorylation in (**D**); (**H**) Western blot of the protein levels of GSK3β and p-GSK3β of Control, GSK3β-OE and Inoistol + GSK3β-OE group. GAPDH was used as loading Control; (**I**–**K**) quantification result of GSK3β and its phosphorylation in (**H**); (**L**) Western blot of the protein levels of β-catenin and CyclinD1 of Control, si-GSK3β, Li_2_CO_3_ + si-GSK3β and Inositol-free + si-GSK3β group; (**M**,**N**) quantification result of β-catenin and CyclinD1 in (**L**); (**O**): Western blot of the protein levels of β-catenin and CyclinD1 of Control, GSK3β-OE and Inoistol + GSK3β-OE group. β-actin was used as a loading Control; (**P**,**Q**) quantification results of β-catenin and CyclinD1 in (**O**). * *p* < 0.05, ** *p* < 0.01 vs. Control group. ^#^ *p* < 0.05, ^##^ *p* < 0.01 vs. si-GSK3β group, ^&&^ *p* < 0.01 vs. GSK3β-OE group. Data are shown as mean ± SD (*n* = 3).

To explore the role of Li_2_CO_3_ on the GSK3β/β-catenin/CyclinD1 pathway via inositol, GSK3β-knockdown and GSK3β-overexpressing mNSC models were established. In GSK3β knockdown [small interfering (si)-GSK3β] cells, both cell viability and proliferation were reduced. Conversely, GSK3β overexpression (GSK3β-OE) cells exhibited enhanced cell viability and proliferation. (Figure 8A,B). The si-GSK3β and GSK3β-OE cells were treated with 1.5 mM Li_2_CO_3_ and 80 mM inositol, respectively, and then qRT-PCR was used to determine the GSK3β mRNA level in each group. As compared with those of the Control, lithium markedly increased cell survival and proliferation, whereas inositol treatment resulted in decreased cell survival and proliferation (Figure 8A,B). The mRNA of GSK3β was increased both in lithium-treated si-GSK3β cells and in inositol-treated GSK3β-OE cells (Figure 8C). Western blotting results revealed that inositol treatment suppressed the activation of the GSK3β/β-catenin/CyclinD1 signaling pathway induced by lithium (Figure 8D–G). Based on the aforementioned results, inositol might affect GSK3β post-transcriptional processes, leading to an imbalance in subsequent phosphorylation, which modulates the GSK3β/β-catenin/CyclinD1 pathway (Figure 8C,L–Q).

## 3. Discussion

**Figure 9:**
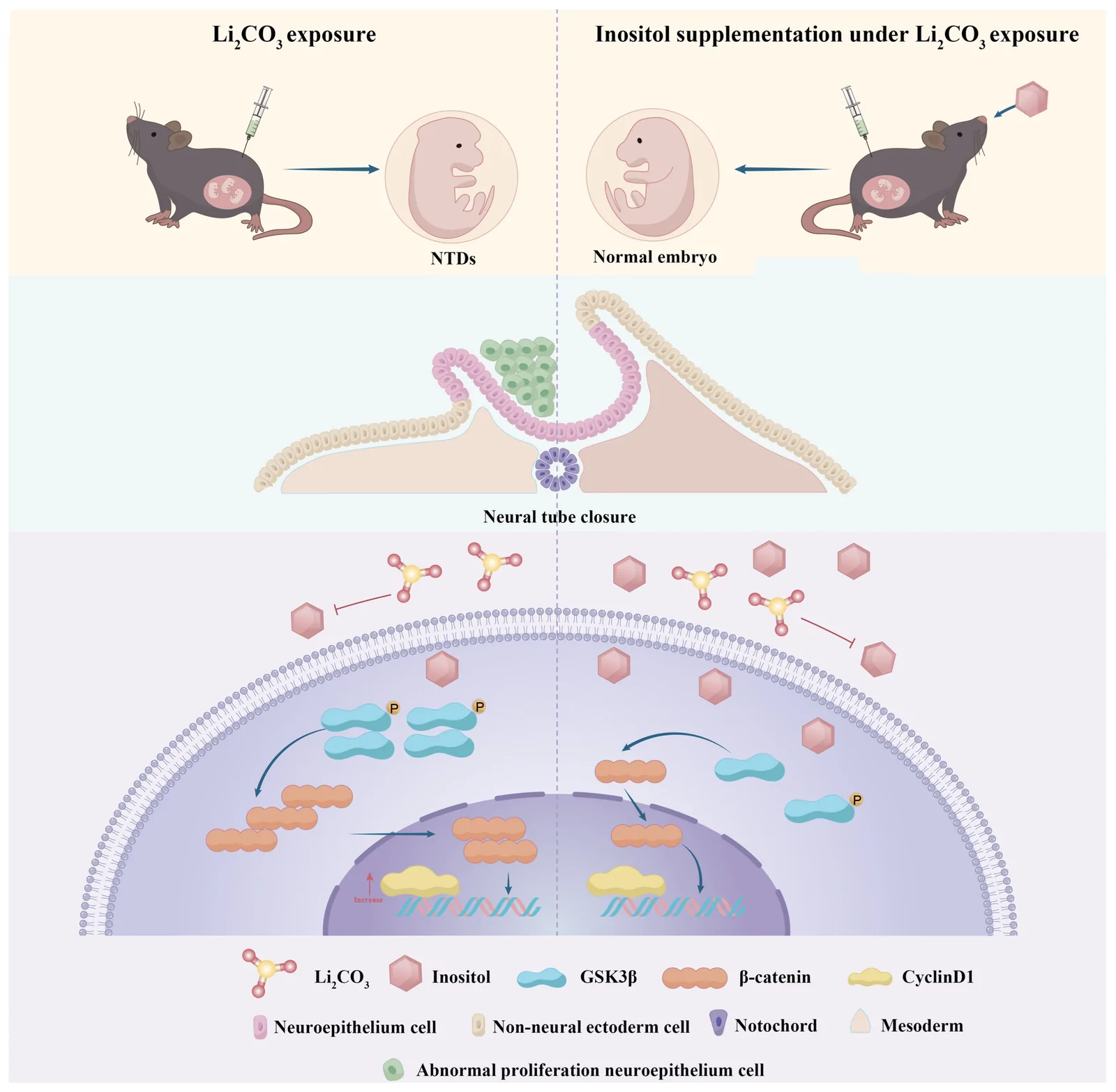
Schematic graph for inositol-dependent alleviation of lithium-induced embryonic neurotoxicity in mice through the GSK3β/β-catenin/CyclinD1 pathway. The process of neural tube closure is illustrated according to [36].

Lithium exposure is toxic for female reproductive health in environmental and fitness contexts [4,5]. The mechanism of Li_2_CO_3_ embryonic neurotoxicity and powerful prevention have not yet been thoroughly studied. The present study revealed that inositol alleviated lithium-prompted embryonic neurotoxicity through the GSK3β/β-catenin/CyclinD1 pathway (Figure 9), which may suggest potential for prevention of BPD in females taking Li_2_CO_3_ within the childbearing age. GSK3β/β-catenin/CyclinD1 pathway is important for numerous neurodevelopmental processes, which include neurogenesis, neuronal migration, neuronal polarization, and axon boom and steering [17]. GSK3β is a key regulator in the coordination of progenitor proliferation and differentiation during brain development [9]. Within the growing mouse neocortex, GSK3β expression was increased in neural progenitors; however, it was often decreased in differentiated neurons [18]. In the present study, increased GSK3β expression was found in normal embryos from E10.5 to E13.5. Exposure to Li_2_CO_3_ increased the levels of GSK3β inhibitory phosphorylation, which activated the β-catenin/CyclinD1 pathway and aberrant cell proliferation, leading to NTDs. Previous studies have shown that lithium promoted neural precursor cell proliferation and neurogenesis by increasing GSK3β/β-catenin/CyclinD1 pathway activity [19,20]. The activation of β-catenin would lead to abnormal cell proliferation [21]. GSK3β inactivation was reported to promote progenitor proliferation [17]. A GSK3β inhibitor elevated the apical lumen length and apical cellular surface area in cortical organoids, which were similar to what is observed in NTDs [22]. Another study confirmed that the inhibition of GSK3β promoted cerebral organoid increase by increasing cell survival and neuronal progenitor proliferation [23]. In addition, antidepressants act through inhibition of GSK3β in the brain via the Ser9 mechanism. The findings of the aforementioned studies are in agreement with those of the present study.

Research indicates that natural compounds can effectively prevent and alleviate the neurotoxic, hepatotoxic, pneumotoxic, nephrotoxic and reproductive toxicity induced by pharmaceuticals in the human body [24,25,26,27]. As a type of natural compound, inositol is present in all animal tissues, with relatively high concentrations observed in the central nervous system. Research suggests that inositol levels are associated with diseases involving brain processes. A reduction in inositol levels is observed in the maternal blood and embryonic brain tissues of NTD pregnancies [28,29]. The brain inositol level is substantially lower in fetuses with hydrocephalus [28]. In the present study, decreased inositol levels were observed under Li_2_CO_3_ exposure, and inositol supplementation alleviated the phenotype of lithium-induced NTDs. The ingestion of inositol can also play a pivotal role in the prevention of NTDs [22]. The PONTI pilot trial reported zero NTD recurrence in high-risk women receiving combined inositol and folic acid versus one recurrence in the folic-acid-only group, supporting inositol as a promising periconceptional supplement [30]. Our previous study found that maternal Li_2_CO_3_ exposure resulted in impaired inositol synthesis in the blood and brain of pregnant mice and disturbed phosphatidylinositol metabolism in embryonic neural tissues [10]. In addition, inositol supplementation partially rescued ciliogenesis impairment, which was associated with neural development, in lithium-treated NIH3T3 cells [4]. Research on inositol and its metabolites focused on potential therapeutic interventions to mitigate the adverse effects of lithium therapy [23]. Furthermore, inositol represents a promising class of drugs for the treatment of psychiatric disorders [31]. The toxicity prediction in the present study also revealed the safety of inositol supplementation.

The present study also found that inositol supplementation reduced lithium-induced abnormal cell proliferation and activated the GSK3β/β-catenin/CyclinD1 pathway. Numerous studies have shown that Li_2_CO_3_ directly inhibits GSK3β. The concentration of Li^+^, which directly inhibited GSK3β in vivo, reportedly exceeded the normal concentration in blood, suggesting that direct GSK3β inhibition might not be the primary mode of action under physiological conditions. The previous finding in pregnant mice exposed to Li_2_CO_3_ indicated that the decline in GSK3β enzyme activity was less pronounced than that of IMPase during neural tube closure, and we hypothesize that Li_2_CO_3_ might not act by directly inhibiting GSK3β [10]. Lithium could also indirectly inhibit GSK3β by activating Akt kinase or by disrupting the β-arrestin complex [32]. The present study revealed that inositol depletion might impair autophagy inside the developing neural tube through the upregulation of the PI3K/Akt pathway [11]. It was reported that d-chiro-inositol decreases the protein expression level of GSK3β in the liver tissue of type 2 diabetic db/db mice through the PI3K/Akt signaling pathway [33]. Our results indicated a binding interaction between GSK3β and inositol with a dose-dependent response. Both animal and cell experimental results indicated that inositol levels modulate GSK3β expression and its phosphorylation. In addition, inositol metabolites were implicated in the regulation of GSK3β. IP5 inhibited GSK3β activity in a dose-dependent manner in mouse F9 teratocarcinoma cells [34]. IP6 was also shown to downregulate GSK3β phosphorylation in a rat colorectal cancer model [35].

To further study how inositol regulates the GSK3β/β-catenin/CyclinD1 pathway, Inositol-free medium and inositol were used to treat si-GSK3β cells and GSK3β-OE cells, respectively. The results demonstrated that GSK3β mRNA levels increased after inositol treatment, whereas GSK3β protein and inhibitory phosphorylation levels decreased significantly. One explanation for this finding might be that inositol affects GSK3β post-transcriptional processes such as mRNA stability, translational efficiency, and protein degradation. These processes could lead to an imbalance in subsequent phosphorylation, which modulates GSK3β enzyme activity. The results of molecular docking and dynamics in the present study revealed the affinity and probable binding mode of the GSK3β-inositol interaction. The mechanism of GSK3β-inositol interaction should be investigated in future experimental studies. The present study clarified the role of inositol in lithium-induced NTDs and preliminarily explored the way inositol acted on the GSK3β/β-catenin/CyclinD1 pathway, laying the foundation for the prevention of lithium-induced embryonic neurotoxicity.

## 4. Materials and Methods

### 4.1. Animals and Treatments

The lithium-induced murine NTD model was established following a previously reported method [10]. The animal experiments were carried out in compliance with the requirements mentioned by the National Institutes of Health Animal Care Standards for the care and use of laboratory animals. All experimental protocols were approved by the Ethics Committee of the Capital Institute of Pediatrics (approval no. DWLL2024020). In brief, C57BL/6J mice, aged 6–8 weeks, weighing 17–19 g, were housed in an experimental animal facility under SPF conditions to limit any possible contamination or health issues. Before commencing experiments, the mice underwent an acclimatization period of at least 2 days to reduce pressure and ensure solid baseline conditions. The mice (female and male) were mated in pairs (1:1 ratio) at 6:00 PM. Vaginal plugs were examined the following morning at 8:00 AM and labeled Embryo day (E) 0.5. The pregnant females were then weighed to screen for any adjustments in body mass related to pregnancy and were randomly assigned into three experimental groups, with 17 mice in the Li_2_CO_3_ group and Control group, and 8 mice in the inositol supplementation group. On E7.5, the experimental group intended for the Li_2_CO_3_ intervention was given an intraperitoneal injection of 350 mg/kg of Li_2_CO_3_ (MilliporeSigma, Burlington, MA, USA); at the same time, the Control was treated with an equal quantity of normal saline. For Li_2_CO_3_-treated mice in the inositol supplementation group, the previous study revealed that maternal plasma inositol levels were lowest 8 h after Li_2_CO_3_ administration [10]. Accordingly, 400 mg/kg myo-inositol (Millipore Sigma) was administered [37] by oral gavage 8 h after Li_2_CO_3_ intervention.

### 4.2. Embryo Examination and Collection

All pregnant mice were anesthetized with 2 min of continuous 3% isoflurane inhalation. Following anesthesia, pregnant mice were euthanized via cervical dislocation, after which the embryos were extracted. The embryos were measured, weighed, and examined for external malformations under a dissecting microscope. Embryos from the normal saline solution and Li_2_CO_3_ intervention groups were collected at E10.5–E13.5 (From E10.5 to 12.5, 3 pregnant mice per group were euthanized daily. On E13.5, 8 pregnant mice per group were sacrificed). Embryos from the inositol supplementation group were collected at E13.5 (8 pregnant mice were sacrificed). The aforementioned collected embryos were divided into three groups: the Control (normal saline solution), NTD (lithium-induced NTDs), and Inositol-sup (normal embryos after inositol supplementation) groups. The embryos were fixed in cold 10% neutral buffered formalin at 4 °C for subsequent immunohistochemical assays. Neural tube tissues from each litter in the Control, NTD, and Inositol-sup embryo groups were isolated and pooled into one sample per group. All the samples were frozen at −80 °C.

### 4.3. Inositol Level Analysis

Neural tissue samples accumulated from murine embryos at E11.5 and E13.5 were aliquoted for myo-inositol detection. A gas chromatography-mass spectrometry (GC-MS) method for the clinical detection of inositol was used as previously established [29].

### 4.4. GSK3β Enzyme Activity Assay

The enzyme activity of GSK3β was determined in embryonic neural tissues through the use of a spectrophotometric quantitative detection package GMS50161.4.3.(GENMED SCIENTIFICS, Arlington, MA, USA) according to the manufacturer’s protocols. Each pattern was analyzed at a minimum of three instances.

### 4.5. Immunohistochemical Assay

Immunohistochemistry was carried out to explore the expression of GSK3β and its phosphorylation in embryonic neural tissues from E10.5 to E13.5. The tissue sections were prepared as described above. After dehydration and blockade of endogenous peroxidase activity, samples were incubated with primary antibodies targeting GSK3β (1:1000; cat. no. 12456, Cell Signaling Technology, Danvers, MA, USA) and phospho-GSK3β (Ser9) (1:40; cat. no. 9323, Cell Signaling Technology) to determine the protein expression levels. To amplify the signal, goat anti-rabbit biotinylated secondary antibody (1:200; cat. no. sc-2774, Santa Cruz Biotechnology, Dallas, TX, USA) was used. After immunostaining, six fields of equal size were randomly selected, and the mean numbers of positive and negative cells were counted under a light microscope. Image-Pro Plus (IPP) 6.0 was used to determine the ratio of positive to negative cells in each image.

### 4.6. Toxicity Analysis and Prediction

Deep-PK is a deep learning-based pharmacokinetic and toxicity prediction, analysis, and optimization platform for small molecules [38], while ADMETlab 3.0 is the second updated version of a prediction platform for exploring the absorption, distribution, metabolism, excretion, and toxicity of compounds in the body [39]. In the present study, these two online systems, Deep-PK (https://biosig.lab.uq.edu.au/deeppk (accessed on 20 May 2025)) and ADMETlab 3.0 (https://admetlab3.scbdd.com (accessed on 20 May 2025)), were applied to analyze the toxicity profiles of Li_2_CO_3_ and inositol using their SMILES notation. These tools provided an in-depth prediction of various toxicological endpoints and pharmacokinetic properties, helping to assess the safety and potential risks associated with lithium carbonate and inositol.

### 4.7. Molecular Docking

The gene targets were retrieved from the Protein Data Bank (PDB) database (https://www.rcsb.org (accessed on 22 June 2025)), and the corresponding target proteins were dwere selected and saved as PDB files. The small compounds were downloaded from the PubChem Compound Database (https://pubchem.ncbi.nlm.nih.gov (accessed on 22 June 2025)) using CAS numbers. The SDF documents containing the 3-D shape were downloaded, and Open Babel was used to convert them to Mol2 format. AutoDock 4.2 and AutoDock gear 1.5.7 were used for molecular docking. The protein was prepared for docking, which involved removing water molecules, adding polar hydrogens, assigning charges, and designating the receptor, followed by conversion to PDBQT format. The small compounds were processed by adding all hydrogens, setting them as the ligands, detecting and setting rotatable bonds, and then saving them in PDBQT format. The ligand compound and protein receptor were then loaded into AutoDock, and the Lamarckian genetic algorithm was used to perform a conformational search. The semiflexible docking method was chosen for molecular docking, and the size of the docking box and grid spacing were set. The conformation with the lowest binding energy was selected, and the complex was visualized using PyMOL 2.3.0 and Ligplot v.1.4.

### 4.8. Molecular Dynamics

To further examine the binding stability of the GSK3β-inositol complex, a molecular dynamics (MD) simulation was completed after molecular docking. The MD simulation mimics the macroscopic surroundings and accounts for factors affecting the binding force between the receptor and ligand. The hundred-nanosecond MD simulations of the complexes were performed via Gromacs 2022 software. Charmm 36 [40] was chosen as the protein force field, the Generation Amber Force Field (Gaff2) was chosen as the ligand force field, and the TIP3P water model was chosen to add solvent to the protein–ligand system and to create a water box with a periodic boundary of 1.2 nm [41]. The particle-mesh Ewald (PME) and Verlet algorithms were used to address electrostatic interactions. Then, 100,000 steps of isothermal isovolumetric ensemble equilibrium and isothermal isobaric ensemble equilibrium were simulated with a coupling constant of 0.1 ps and a duration of 100 ps. Both van der Waals and Coulomb interactions were calculated using a 1.0 nm cutoff value. Finally, the system was simulated using Gromacs 2022 at constant temperature (310 K) and constant pressure (1 bar) for a total of 100 ns. Using the binding conformations of the complexes, the free energy of binding of small molecules to the target proteins was calculated using the Molecular Mechanics/Poisson-Boltzmann Surface Area (MM/PBSA) method. The root mean square deviation (RMSD) was used to observe the local site deformation of the system during the simulation. The radius of gyration (Rg) was used to evaluate the tightness of the system structure. The root mean square function (RMSF) was used to observe the local site deformation of the system during the simulation process. The solvent-accessible surface area (SASA) was used to observe the size of the solvent accessible surface area of the complex during the simulation process.

### 4.9. Surface Plasmon Resonance (SPR)

The experiment was performed on a Biacore T200 instrument (Cytiva, Marlborough, MA, USA). A CM5 sensor chip (Cytiva, Marlborough, MA, USA) was selected for molecular interaction analysis. The assay buffer consisted of 10 mM HEPES, 150 mM NaCl, 3 mM EDTA, and 0.05% P20. The ligand protein (GSK3β, cat. no. HY-P73090, MedChemExpress, Monmouth Junction, NJ, USA) was immobilized onto the chip surface via the Amine Coupling method. Protein samples, diluted in a sodium acetate buffer (pH 4.5), were injected at a flow rate of 10 μL/min to facilitate the immobilization reaction, which was completed using an amine coupling kit (cat. no. BR-1000-50, Cytiva, Marlborough, MA, USA). Test compounds (inositol, MilliporeSigma) were serially diluted to establish eight concentration gradients (0.42, 0.84, 1.7, 3.4, 6.8, 14, 27 and 54 μM). The analyte was passed over the chip at a flow rate of 30 μL/min. The association and dissociation times were each set to a duration of 300 s. Global fitting of the resulting kinetic data was performed using a steady-state binding model.

### 4.10. Cell Culture, Treatments and Transient Transfection

Mouse neural stem cells (mNSCs) of the NE4C cell line (immortalized cells, cat. no. CRL-2925) and mouse embryonic fibroblasts (mEFCs) of the NIH3T3 cell line (immortalized cells; cat. no. CRL-1658) were purchased from the Stem Cell Bank of the Chinese Academy of Sciences. The mEFCs were cultured in DMEM (Gibco; Thermo Fisher Scientific, Grand Island, NY, USA) supplemented with 10% FBS (Gibco; Thermo Fisher Scientific) and 1% penicillin/streptomycin (Gibco; Thermo Fisher Scientific). After starvation, mEFCs were treated with different concentrations of Li_2_CO_3_. Firstly, culture flaps/plates were covered with 15 µg/mL poly-D-lysine (PDL; MilliporeSigma) to help cells adhere. The mNSCs were cultured in MEM (Gibco; Thermo Fisher Scientific) supplemented with 10% FBS (Gibco; Thermo Fisher Scientific), 1% non-essential amino acids (Gibco; Thermo Fisher Scientific), 1% glutamine (Gibco; Thermo Fisher Scientific), and 1% penicillin/streptomycin (Gibco; Thermo Fisher Scientific). After starvation, mNSCs were subjected to exclusive treatments: Control (PBS), Li_2_CO_3_ (1.5 mM), Inositol-free (Inositol-free MEM), High-inositol (80 mM), and Inositol-sup (1.5 mM Li_2_CO_3_ + 80 mM inositol).

Small interfering RNA against GSK3β (si-GSK3β) and its corresponding Control (si-NC) were designed and synthesized by GenePharma (Shanghai, China) and then transiently transfected into mNSCs, which were seeded onto six-well plates using Lipofectamine 2000^®^ (Invitrogen; Thermo Fisher Scientific) for 24 h following the manufacturer’s instructions. Untreated cells were considered to be the Control group. After 24 h of transfection, the cells were treated with 1.5 mM Li_2_CO_3_ and Inositol-free medium for 24 h, respectively.

The CDS region series of the target gene GSK3β was inserted into the pcDNA3.1 vector. The generation of mNSCs overexpressing GSK3β was carried out by transfecting cells at 70–80% confluency with pcDNA3.1-GSK3β (2 μg/well; Shanghai GenePharma, Shanghai, China) using Lipofectamine 2000^®^ according to the manufacturer’s instructions. After 24 h of transfection, the cells were treated with 80 mM inositol for 24 h. The expression levels of GSK3β were detected through quantitative qRT-PCR and Western blotting.

### 4.11. Cell Viability Assay

The mNSCs and mEFCs were seeded and cultured in 96-well plates for 24 h, after which they were treated with specific concentrations (0, 0.5, 1, 1.5, 2, 2.5, 3, 5 and 10 mM) of Li_2_CO_3_ for an additional 24 h. Subsequently, the cells were incubated with medium containing 5 mg/mL MTT (MilliporeSigma) for 4 h. To terminate the reaction, 150 µL of DMSO (Beijing Solarbio Science & Technology, Beijing, China) was added to each well. The absorbance of the plate was then measured at 490 nm using a microplate reader (Thermo Fisher Scientific). The mNSCs were then cotreated with Li_2_CO_3_ (at the concentration producing the highest survival rate) and inositol at concentrations of 0, 0.1, 0.5, 1, 5, 10, 20, 40, 80, 100, and 150 mM to identify the optimal inositol dose.

### 4.12. Cell Cycle Assay

The assay was executed using a cell cycle assay kit (cat. no. CA1510; Beijing Solarbio Science & Technology, Beijing, China) consistent with the manufacturer’s instructions. The concentrations of Li_2_CO_3_ that caused each cell to have the highest survival rate were selected on the basis of the results of the cell viability assay. After 12 h of serum starvation, the mNSCs and mEFCs were subjected to Li_2_CO_3_ for 24 h, and an equal volume of PBS solution was added to the Control group. The cells were harvested, washed twice with PBS, and resuspended in 100 µL of PBS. They were then fixed with 1 mL of 75% ethanol at 4 °C for 4 h. After fixation, the cells were washed, and 100 µL of RNaseA was added to the cell pellet. The mixture was thoroughly mixed and incubated at 37 °C for 30 min. The cells were subsequently stained with propidium iodide, incubated in the dark at 4 °C for 30 min, and filtered through a strainer cap before being analyzed by flow cytometry (Beckman Coulter, Brea, CA, USA). The cell cycle distribution was analyzed using ModFit 5.0 software.

### 4.13. Cell Proliferation Assay

The results of Li_2_CO_3_ on mNSC and mEFC proliferation were evaluated using Clik-iT EdU Imaging Kits (Invitrogen; Thermo Fisher Scientific, Waltham, MA, USA) consistent with the manufacturer’s instructions. A total of six fields of equal size were randomly selected, and the number of positive cells was counted.

### 4.14. Immunofluorescence Assay

To determine the expression of the GSK3β/β-catenin/CyclinD1 pathway, mNSCs were labeled with GSK3β (1:200; cat. no. 12456; Cell Signaling Technology), phospho-GSK3β (Ser9; 1:100; cat. no. 9323; Cell Signaling Technology), β-catenin (1:200; cat. no. 8480; Cell Signaling Technology) and CyclinD1 (1:200; cat. no. 60186-1-Ig; Proteintech Group, Rosemont, IL, USA). Then, a secondary antibody (1:1000; cat. no. 4414; cat. no. 4408; Cell Signaling Technology, Danvers, MA, USA) and DAPI-containing mounting medium (cat. no. ab104139; Abcam, Cambridge, UK) were used for fluorescence staining. Photomicrographs were acquired with a confocal microscope (SP8, Leica Microsystems, Wetzlar, Germany) and the images were analyzed with ImageJ 1.54p (National Institutes of Health).

### 4.15. Quantitative Real-Time Polymerase Chain Reaction (qRT-PCR) and Western Blot Analysis

Overall RNA extraction and cDNA synthesis from the embryonic brain tissues and mNSCs were carried out using RNeasy R Micro kit (Qiagen China, Shanghai, China) and TransScript First-Strand cDNA Synthesis SuperMix (TransGen Biotech, Beijing, China). qRT-PCR was performed using a qPCR MasterMix Kit (Applied Biological Materials, Richmond, BC, Canada), and fluorescence was detected using a QuantStudio 7 Flex (Applied Biosystems; Thermo Fisher Scientific, Waltham, MA, USA). The following sequences were used: GSK3β: Forward primer (5′-ACACCTGCACTCTTCAACTTTACC-3′) and reverse primer (5′-GTTATTGGTCTGTCCACGGTCTC-3′); GAPDH: Forward primer (5′-CGTCCCGTAGACAAAATGGT-3′) and reverse primer (5′-GAATTTGCCGTGAGTGGAGT-3′); ACTB: Forward primer (5′-AAGATCAAGATCATTGCTCCTCC-3′) and reverse primer (5′-GACTCATCGTACTCCTGCTTGC-3′). The expression degrees of mRNA were calculated with the 2-ΔΔCq method [42]. Gene expression of target proteins was normalized to GAPDH or ACTB expression.

Protein samples extracted from neural tissues and mNSCs were separated by SDSPAGE page. Western blotting evaluation was carried out as previously described [43]. Antibodies against PH3 (1:400; cat. no. 9701; Cell Signaling Technology), PCNA (1:600; cat. no. 13110; Cell Signaling Technology), GSK3β (1:1000; cat. no. 12456; Cell Signaling Technology), phospho-GSK3β (Ser9; 1:600; cat. no. 9323; Cell Signaling Technology), β-catenin (1:1000; cat. no. 8480; Cell Signaling Technology), CyclinD1 (1:2000; cat. no. 60186-1-Ig; Proteintech Group), GAPDH (1:1000; cat. no. 5174; Cell Signaling Technology) and β-actin (1:1000; cat. no. 4970; Cell Signaling Technology) were used. GAPDH and β-actin were used as internal Controls.

### 4.16. Statistical Analysis

Data are expressed as the mean ± standard deviation. The differences between the two groups were analyzed by unpaired Student’s *t* tests. The groups were compared via one-way ANOVA followed by Dunnett’s post hoc test for multiple comparisons. A *p* < 0.05 was considered to indicate a statistically significant difference. Analyses were performed using SPSS version 30.0 software (IBM Corp., Armonk, NY, USA). GraphPad Prism 10 software (Dotmatics, Boston, MA, USA) was used for graphing.

## 5. Conclusions

Inositol alleviated lithium-prompted embryonic neurotoxicity via the GSK3β/β-catenin/CyclinD1 pathway and preliminarily explored how inositol acted on the GSK3β/β-catenin/CyclinD1 pathway, laying the groundwork for further research to clarify the mechanisms underlying lithium-induced neurotoxicity. Inositol has potential as a therapeutic prevention method for lithium-induced embryonic neurotoxicity for female patients of childbearing age with BPD.

## Figures and Tables

**Table 1:** The results of repeated experiments of NTD model establishment at E13.5.

Li_2_CO_3_ mg/kg	Inositol mg/kg	Litters *n*	Embryos *n*	Normal *n* (%)	Resorption *n* (%)	NTDs *n* (%)	Other Malformations *n* (%)	*p*-Value
0	0	8	72	72 (100)	0 (0)	0 (0)	0 (0)	<0.01
350	0	8	63	13 (20.6)	5 (7.9)	21 (33.3)	14 *; 2 ^#^; 2 ^&^; 6 ^§^ (38.2)	

* Eye malformations include microphthalmia and anophthalmia; ^#^ facial malformation; ^&^ craniofacial malformation; ^§^ growth retardation.

**Table 2:** Toxicity prediction of Li_2_CO_3_ and inositol.

Property	ADMETlab				Deep-PK			
	Li_2_CO_3_		Inositol		Li_2_CO_3_		Inositol	
	Value	Decision	Value	Decision	Value	Decision	Value	Decision
Toxicity								
hERG Blockers	0.016	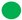	0.070	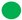	0.092	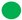	0.352	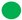
Drug-induced Liver Injury	0.188	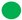	0.017	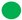	0.999	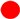	0.265	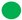
Rat Oral Acute Toxicity	0.275	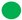	0.141	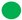	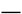	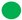	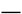	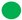
Skin Sensitization	0.983	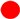	0.001	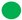	0.376	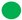	0.768	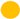
Carcinogenicity	0.627	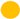	0.078	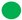	0.977	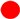	0.172	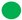
Eye Corrosion	0.983	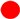	0.000	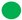	1	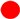	0	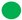
Eye Irritation	0.999	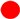	0.108	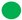	1	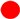	0.011	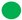
Respiratory	0.230	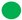	0.002	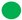	0.453	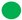	0.007	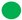
Human Hepatotoxicity	0.893	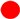	0.208	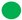	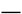		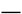	
Drug-induced Nephrotoxicity	0.119	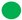	0.042	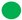	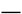		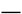	
Hematotoxicity	0.568	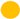	0.011	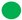	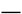		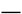	
Genotoxicity	1.000	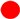	0.000	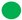	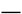		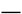	
RPMI-8226 Immunitoxicity	0.014	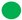	0.015	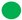	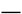		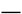	
A549 Cytotoxicity	0.004	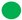	0.006	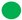	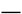		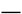	
Hek293 Cytotoxicity	0.006	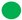	0.115	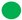	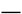		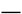	
Drug-induced Neurotoxicity	0.525	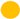	0.077	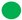	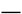		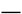	
Micronucleos	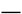		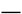		1.000	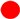	0.002	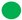
Tox21 pathway								
NR-AhR	0.055	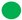	0.000	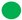	0.000	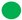	0.000	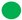
NR-AR	0.027	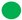	0.000	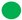	0.106	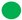	0.013	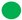
NR-AR-LBD	0.002	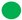	0.000	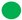	0.000	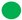	0.023	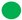
NR-Aromatase	0.003	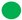	0.000	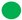	0.000	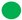	0.000	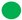
NR-ER	0.074	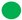	0.030	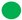	1	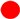	0.071	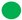
NR-ER-LBD	0.005	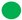	0.000	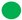	0.000	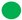	0.004	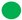
NR-PPAR-gamma	0.003	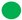	0.000	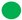	0.000	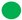	0.000	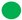
SR-ARE	0.025	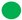	0.000	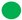	0.000	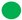	0.039	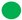
SR-ATAD5	0.000	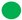	0.000	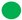	0.000	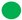	0.000	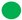
SR-HSE	0.001	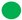	0.000	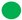	0.000	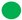	0.000	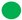
SR-MMP	0.001	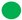	0.000	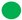	0.000	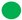	0.000	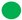
SR-p53	0.002	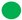	0.000	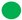	0.000	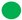	0.001	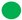

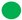
 Safe; 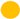 Medium; 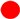 Toxic; 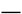 No mention. NR: Nuclear receptors; AhR: Aryl hydrocarbon receptor; AR: Androgen receptor; LBD: Ligand-binding domain; ER: estrogen receptor; PPAR: Peroxisome proliferator-activated receptor. SR: Sensitivity ratio; ARE: Antioxidant response element; ATAD5: ATPase family AAA domain-containing protein 5; HSE: Heat shock factor response element; MMP: Mitochondrial membrane potential; p53: a tumor suppressor protein.

**Table 3:** Lithium-induced NTD murine model with inositol supplementation on E13.5.

Li_2_CO_3_ mg/kg	Inositol mg/kg	Litters *n*	Embryos *n*	Normal *n* (%)	Resorption *n* (%)	NTDs *n* (%)	Other Malformations *n* (%)	*p*-Value
0	0	8	72	72 (100)	0 (0)	0 (0)	0 (0)	<0.01
350	0	8	63	13 (20.6)	5 (7.9)	21 (33.3)	14 *; 2 ^#^; 2 ^&^; 6 ^§^ (38.2)	
350	400	8	74	45 (60.8)	1 (1.4)	11 (14.9)	14 *; 2 ^#^; 1 ^&^ (22.9)	

* Eye malformations include microphthalmia and anophthalmia; ^#^ facial malformation; ^&^ craniofacial malformation; ^§^ growth retardation.

## Data Availability

The original contributions presented in this study are included in the article. Further inquiries can be directed to the corresponding author.

## Abbreviations

The following abbreviations are used in this manuscript:

Abbreviation	Full term
Li_2_CO_3_	Lithium carbonate
BPD	Bipolar disorder
NTDs	Neural tube defects
GSK3β	Glycogen synthase kinase 3β
CyclinD1	Cell cycle protein D1
E 0.5	Embryo day 0.5
GC-MS	Gas chromatography-mass spectrometry
PDB	Protein Data Bank
MD	Molecular dynamics
MM/PBSA	Molecular Mechanics/Poisson-Boltzmann Surface Area
RMSD	Root mean square deviation
Rg	Radius of gyration
RMSF	Root mean square function
SASA	Solvent-accessible surface area
SPR	Surface plasmon resonance
mNSCs	Mouse neural stem cells
mEFCs	Mouse embryonic fibroblasts
PDL	Poly-D-lysine
qRT-PCR	Quantitative real-time polymerase chain reaction
GAPDH	Glyceraldehyde 3-phosphate dehydrogenase
PH3	Phosphohistone H3
PCNA	Proliferating cell nuclear antigen
SDs	Standard deviations
CRL	Crown-rump length
